# THE IMPACT OF DEMENTIA TRAINING ON HOSPITAL STAFF’S KNOWLEDGE AND ATTITUDES

**DOI:** 10.1093/geroni/igz038.2664

**Published:** 2019-11-08

**Authors:** Julia Schneider, Birgit Teichmann, Andreas Kruse

**Affiliations:** 1 Network Aging Research, University of Heidelberg, Heidelberg, Baden-Wurttemberg, Germany; 2 Institute of Gerontology, University of Heidelberg, Heidelberg, Baden-Wurttemberg, Germany

## Abstract

Older people with dementia are more frequently hospitalized and more strongly associated with negative outcomes. We examined the acceptance and the effect of a dementia training on attitudes and knowledge of the hospital staff. In the current study, we utilized a mixed-methods research design to examine a clinic group of six hospitals in Germany. Besides semi-structured interviews, we collected quantitative data with a questionnaire given before and three-months after the training. The questionnaire contained German translated versions of the Knowledge in Dementia (KIDE) Scale and the Dementia Attitudes Scale (DAS-D) to assess changes in attitudes. The participant population (N=60) consisted of nurses (n=35, 58%), medical assistants (n=13, 22%) and other medical professions. Satisfaction with the training was predominantly positive, 92% would recommend the training to their colleagues. At baseline, a small but significant correlation between the standardized questionnaires KIDE and DAS-D was evident (r(60)=.357, p=.005). The participants (n=32) showed a more positive attitude in the post-test (M=5.39, SD=0.64) than in the baseline-test (M=5.19, SD=0.66). This difference was significant (t(31)=-2.434, p=.021). However, we did not find any significant effects on the KIDE. The reason for this may be the use of a standardized questionnaire, which does not reflect the increase in knowledge, or there has been no increase in knowledge of dementia. The results are based on a small sample size. However, they have demonstrated that dementia training can positively influence attitudes toward people with dementia. A significant increase in knowledge was expected but could not be demonstrated.

